# Augmented reality–assisted craniofacial reconstruction in skull base lesions — an innovative technique for single-step resection and cranioplasty in neurosurgery

**DOI:** 10.1007/s10143-022-01784-6

**Published:** 2022-04-20

**Authors:** Christine Steiert, Simon Phillipp Behringer, Luisa Mona Kraus, Marco Bissolo, Theo Demerath, Juergen Beck, Juergen Grauvogel, Peter Christoph Reinacher

**Affiliations:** 1 Department of Neurosurgery, Medical Center – University of Freiburg, Faculty of Medicine, University of Freiburg, Freiburg, Germany; 2 Department of Neuroradiology, Medical Center – University of Freiburg, Faculty of Medicine, University of Freiburg, Freiburg, Germany; 3 Department of Stereotactic and Functional Neurosurgery, Medical Center - University of Freiburg, Faculty of Medicine, University of Freiburg, Freiburg, Germany; 4 Fraunhofer Institute for Laser Technology, Aachen, Germany

**Keywords:** Augmented reality, Mixed reality, Skull base, Cranioplasty, Craniofacial reconstruction

## Abstract

Defects of the cranial vault often require cosmetic reconstruction with patient-specific implants, particularly in cases of craniofacial involvement. However, fabrication takes time and is expensive; therefore, efforts must be made to develop more rapidly available and more cost-effective alternatives. The current study investigated the feasibility of an augmented reality (AR)–assisted single-step procedure for repairing bony defects involving the facial skeleton and the skull base. In an experimental setting, nine neurosurgeons fabricated AR-assisted and conventionally shaped (“freehand”) implants from polymethylmethacrylate (PMMA) on a skull model with a craniofacial bony defect. Deviations of the surface profile in comparison with the original model were quantified by means of volumetry, and the cosmetic results were evaluated using a multicomponent scoring system, each by two blinded neurosurgeons. Handling the AR equipment proved to be quite comfortable. The median volume deviating from the surface profile of the original model was low in the AR-assisted implants (6.40 cm^3^) and significantly reduced in comparison with the conventionally shaped implants (13.48 cm^3^). The cosmetic appearance of the AR-assisted implants was rated as very good (median 25.00 out of 30 points) and significantly improved in comparison with the conventionally shaped implants (median 14.75 out of 30 points). Our experiments showed outstanding results regarding the possibilities of AR-assisted procedures for single-step reconstruction of craniofacial defects. Although patient-specific implants still represent the gold standard in esthetic aspects, AR-assisted procedures hold high potential for an immediately and widely available, cost-effective alternative providing excellent cosmetic outcomes.

**Supplementary Information:**

The online version contains supplementary material available at 10.1007/s10143-022-01784-6.

## Introduction

Cosmetic reconstructions are usually required after surgical resection of bony lesions of the skull, particularly in frontal localization or involvement of the facial skeleton and the skull base [4, 27]. While the previously common use of prefabricated titanium implants or intraoperatively shaped implants (e.g., polymethylmethacrylate (PMMA)) did not always provide satisfactory cosmetic results, these have been markedly improved by the availability of patient-specific (computer-assisted design/manufacturing (CAD/CAM)) implants [1, 3, 14]. These originally always required a two-stage surgical procedure, as the implant first had to be manufactured after the tumor resection [7, 13]. Due to current technical developments, it is now increasingly possible to fabricate the implant in advance on the basis of a virtually performed resection so that only one operation is necessary for resection and reconstruction [8, 24, 38]. This procedure seems to be particularly suitable for benign lesions and is described in some reports for sphenoorbital meningiomas [5, 10, 28]. However, the relatively high costs of patient-specific implants must be considered, which makes them unlikely to be affordable to a broad majority worldwide [15, 20]. Furthermore, due to the typically several weeks required for commercial implant fabrication, this single-step procedure is not feasible for aggressive or malignant lesions, and, in addition, not an option for acute reconstruction after trauma or when intraoperative adjustment of resection margins is required.

The implementation of augmented reality (AR) promises new possibilities in this area. With regard to applications in cranial neurosurgery, various AR-based simulations for cerebrovascular, brain tumor, and skull base procedures have been described so far [2, 11, 18, 31, 39], as have AR-supported training concepts in brain tumor and skull base surgery [16, 19, 32]. As for intraoperative use, descriptions of AR-guided navigation can be found primarily for endoscopic procedures, including endoscopic transsphenoidal skull base surgery [9, 25, 29]. Several phantom studies also demonstrate potential applications for cranial biopsy or catheter placement [33, 36].

The present study is the first to investigate the feasibility of AR-assisted reconstruction of bony defects involving the facial skeleton and the skull base for potential application in affected patients.

## Materials and methods

### Study design

**Fig. 1:**
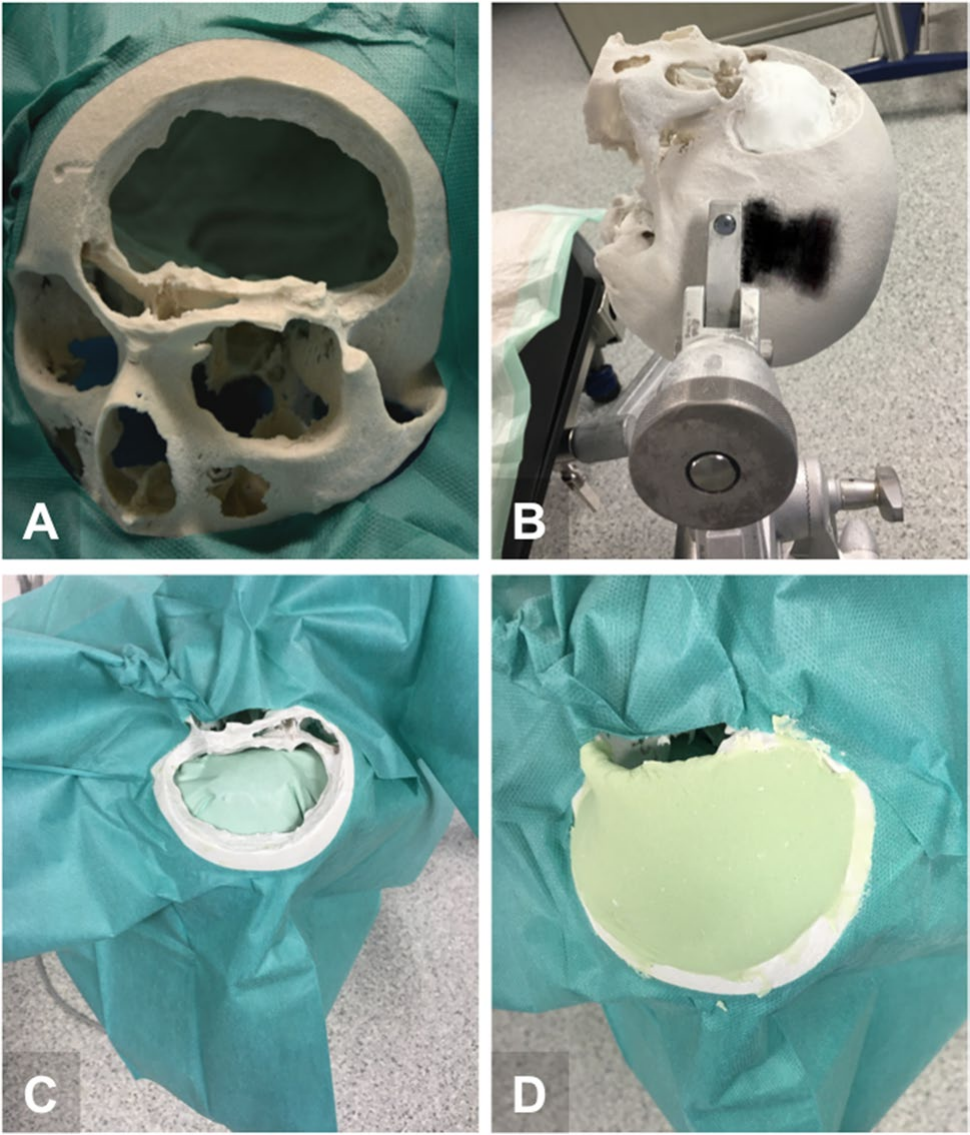
Experimental setup for implant fabrication. (**A**) Insertion of the defect into the original skull model; (**B**) clamp-fixation of the model, packing with cottonoids to simulate the surface of the dura; (**C**) covering of the surrounding surface to simulate the intraoperative situation; (**D**) adaptation of the PMMA implant for defect repair

A computed tomography (CT) scan (SOMATOM® Definition AS, Siemens Healthcare GmbH, Erlangen, Germany) of an x-ray dense skull model was performed (tube voltage 120 kV, exposure 74 mAs, gantry tilt 0°, slice thickness 0.75 mm, helical mode), serving as ground truth. The three-dimensional (3D) reconstructed scan was transferred to the AR device (Magic Leap 1, Magic Leap, Plantation, Florida). Thereafter, a defect involving the frontal skull and orbital rim was inserted into the model, with localization and extension corresponding to common pathologies of the anterior skull base involving the orbital rim (e.g., sphenoorbital meningiomas, fibrous dysplasia, calvarial metastases). Subsequently, nine participants (three experienced neurosurgeons and six less experienced younger neurosurgery residents) each produced two AR-assisted and two conventionally shaped (“freehand”) implants from PMMA (Palacos®R + G, Heraeus Medical GmbH, Wehrheim, Germany) for defect repair. AR assistance was provided using Magic Leap 1 combined with Brainlab® Mixed Reality Viewer (Brainlab AG, Munich, Germany) by manually positioning the holographic projection of the original skull model (ground truth) over the real model with the inserted defect. To simulate the real intraoperative situation, the clamp-fixated skull model was covered after AR alignment (except for the defect to be repaired), as shown in Fig. 1. With the AR-assisted procedure, both the preparation of the situs by layering with cottonoids according to the convexity and the modeling of PMMA before hardening were performed under AR-guidance (see Video 1). The order of the participants and the type of implant fabrication were randomized (random.org, Dublin, Ireland). For both the conventionally shaped (“freehand”) implants and the AR-assisted implants, there was only one trial per implant, and in both groups there was no subsequent optimization after hardening, e.g., by adding PMMA or drilling protruding material. The finished implants were pseudonymized and a subsequent CT scan was performed of each after insertion into the original skull model. Participants’ subjective impressions of manageability during the fabrication process and satisfaction with the results were evaluated with a questionnaire (Table 1). Ethical approval was not required because this was a pure phantom study.

**Table 1:**
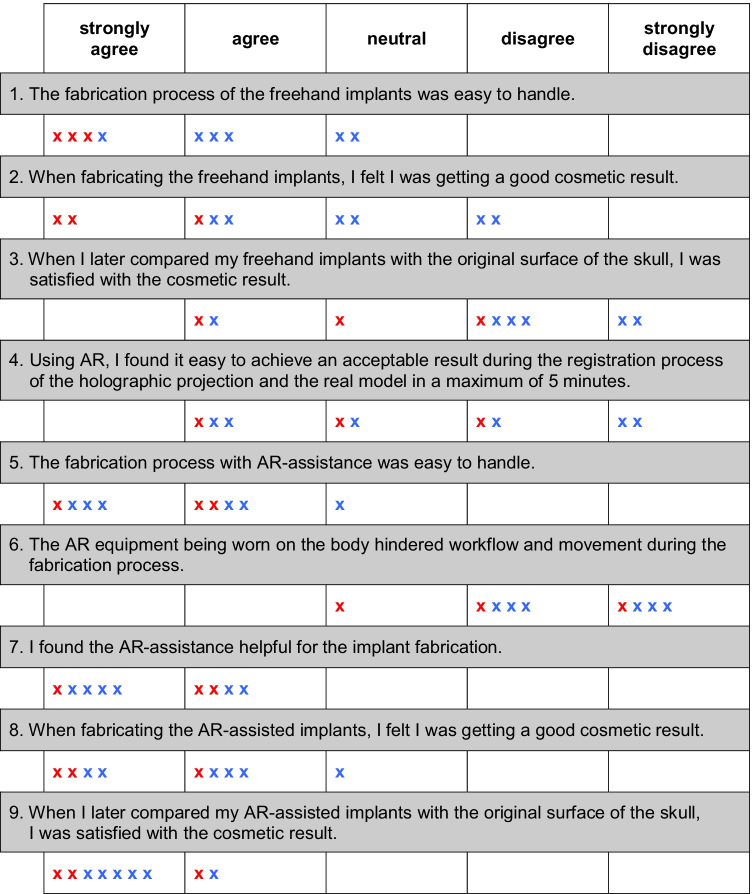
Questionnaire for participants’ evaluation and survey results

Based on a Likert scale, participants evaluated the fabrication process, the handling of the AR equipment and their satisfaction with the (cosmetic) results (one answer per question per participant). x (red), answer of experienced neurosurgeons; x (blue), answer of less experienced neurosurgeons

### Volumetric analysis

**Fig. 2:**
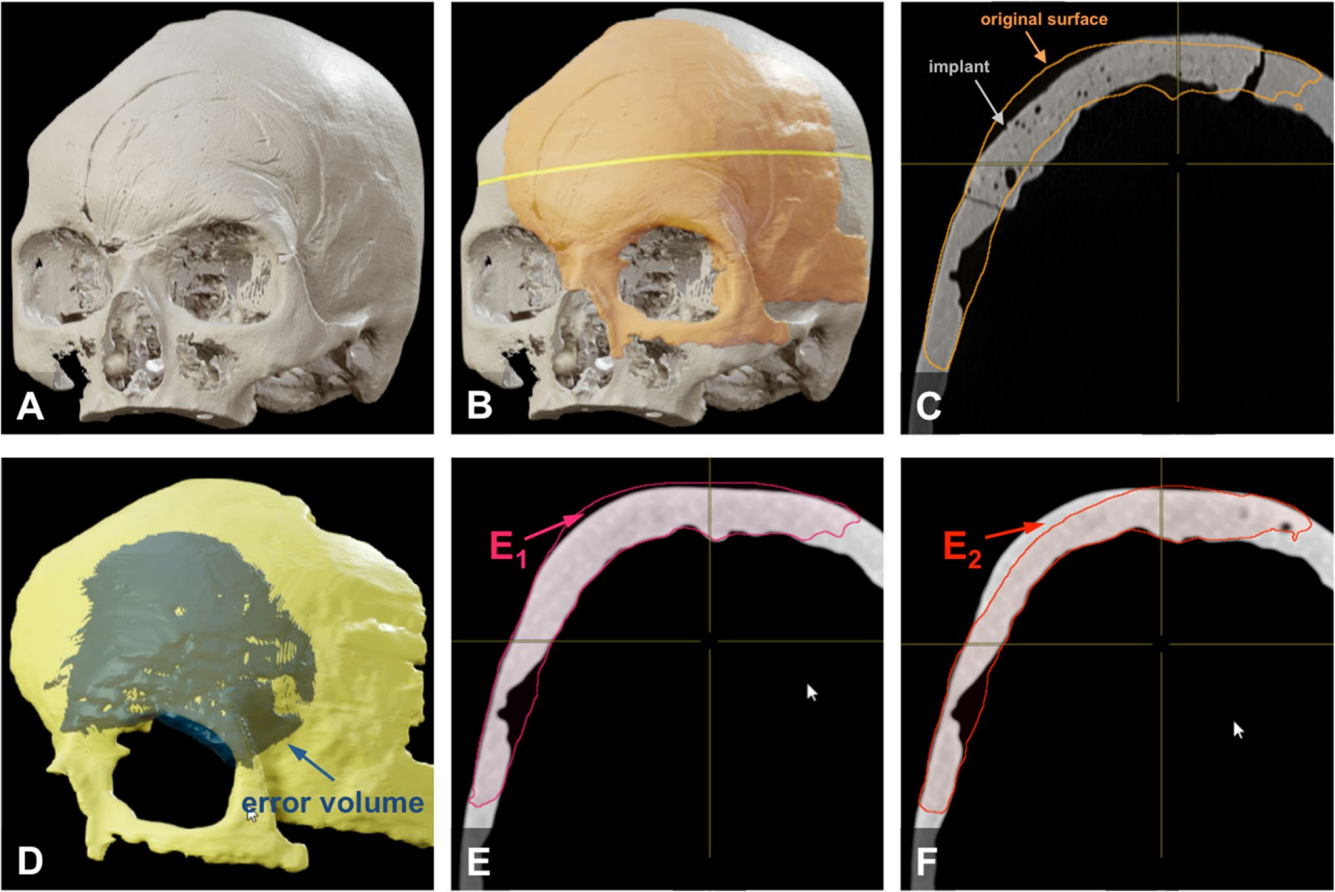
Schematic illustration of the evaluation of volumetric results. (**A**) 3D-reconstructed CT scan of the skull model with an inserted PMMA implant; (**B**) projection of a part of the surface of the original skull model (orange) onto the model with the inserted implant (yellow line: cross section shown in image C); (**C**) cross section from image B showing the projection of the surface of the original skull model (orange) and the surface of the implant; (**D**) 3D-reconstructed part of the surface of the original skull model (yellow) with the overlying projection of the deviating error volume *E*_total_ of an implant (blue); (**E**) cross section of the surface of the original skull model with the overlying projection of an implant (pink), example of an overlapping volume *E*_1_; (**F**) cross section of the surface of the original skull model with the overlying projection of an implant (red), example of a lacking volume *E*_2_

Volumetric deviations of the surface profiles of the implants in comparison with the original skull model were each quantified by two independent, blinded investigators using the SmartBrush device on Brainlab Origin Server 3.2 (Brainlab AG, Munich, Germany), and the corresponding mean values were used for further analysis. Both the overlapping (error volume *E*_1_) and the lacking (error volume *E*_2_) volume of the implants in comparison with the surface profile of the original model were measured, as illustrated in Fig. 2. The total error volume was calculated from the sum of *E*_1_ and *E*_2_ (error volume *E*_total_).

### Evaluation of cosmetic results

**Fig. 3:**
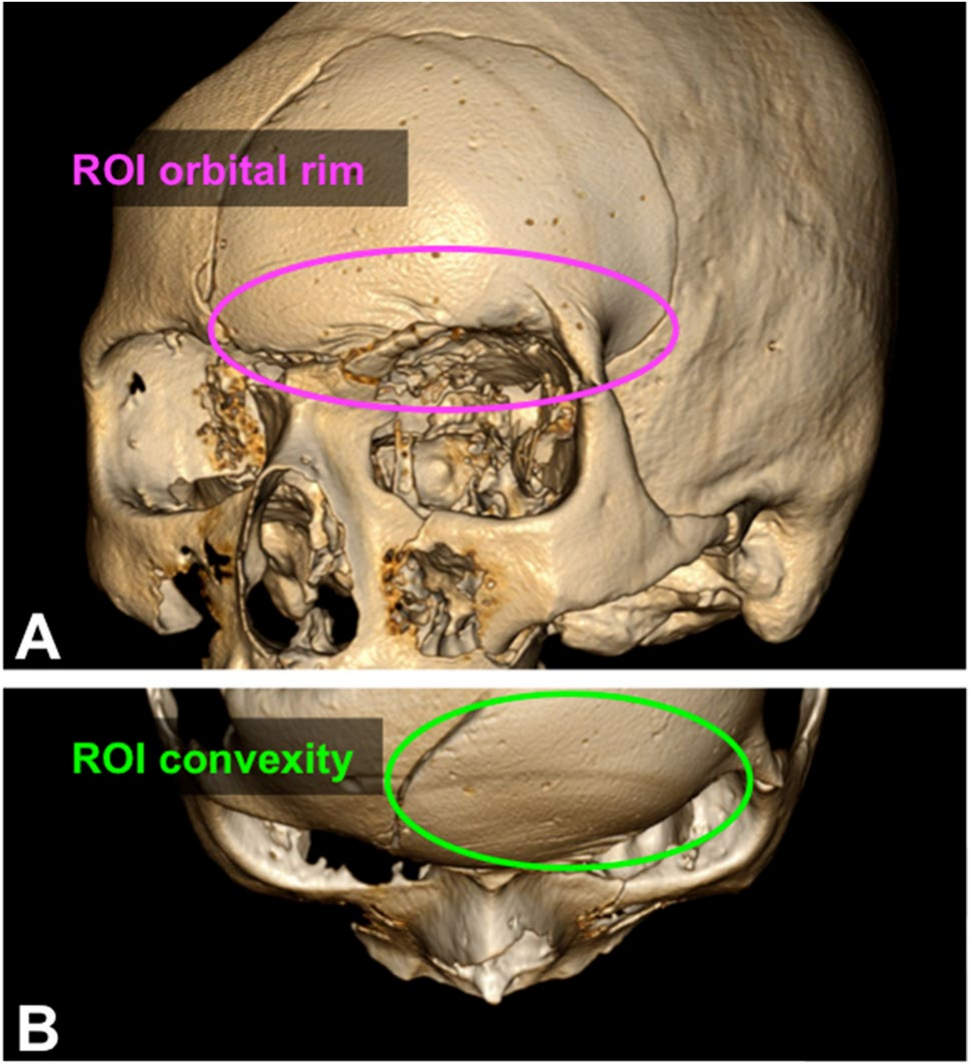
Illustration of the specific ROIs for the evaluation of cosmetic results. (**A**) 3D-reconstructed CT scan of the skull model with an inserted PMMA implant showing the area of the ROI reconstruction of the orbital rim (pink, ROI orbital rim); (**B**) 3D-reconstructed CT scan of the skull model with an inserted PMMA implant showing the area of the ROI convexity of the frontal tuberosity (green, ROI convexity)

The cosmetic results of the AR-assisted and the conventionally shaped implants were compared with the original skull model. For this purpose, the implants were each evaluated independently by two neurosurgeons in a blinded fashion (based on the pseudonymized implants and the corresponding 3D-reconstructed CT scans). The mean values were used for further analysis. Points between 0 and 10 were awarded for the overall cosmetic appearance and for two specific regions of interest (ROIs) (reconstruction of the orbital rim and convexity of the frontal tuberosity, as illustrated in Fig. 3). Thus, the highest score to be achieved was 30.

### Statistical analysis

Methods of descriptive statistics were used. For numerical data, median values and the interquartile range (IQR) were calculated. Interrater reliability in volumetric analysis and cosmetic scoring was validated using a Bland–Altman plot. Statistical differences were evaluated using a Mann–Whitney test as an unpaired nonparametric test (data were not normally distributed upon testing). The level of significance was set to *p* < 0.05. Statistical analysis was performed using GraphPad Prism software version 9.1.0 for Mac (GraphPad Software, San Diego, CA, USA).

## Results

### AR equipment handling

The AR hardware and software were easy to use for both experienced and less experienced neurosurgeons in the operating theater. The manual registration process (positioning the holographic projection over the real model) took 5–10 min, depending on the individual experience. The well-fitting AR glasses allowed free head movement and the associated equipment was small and could be easily worn on the body. The battery life was adequate, and individual adjustment of the light made it possible to vary the translucency of the image in the glasses to see more of the real image or the AR projection as required. The surface profile of the holographic object was well visualized, even in a complex region. By altering the distance of the glasses to the object, the image was also displayed in slices, like scrolling through a CT scan, which was additionally helpful. After the registration process, the time needed for implant fabrication (preparation of the situs with cottonoids and PMMA fitting) was measured. This procedure took 10–20 min, depending on the experience of the neurosurgeon, with no relevant difference between the AR-assisted and the conventionally shaped implants. The AR-assisted fabrication process is demonstrated in Video 1. Participants’ detailed evaluation of the fabrication process and their satisfaction with the results based on the questionnaire are shown in Table 1.

### Volumetric analysis

**Fig. 4:**
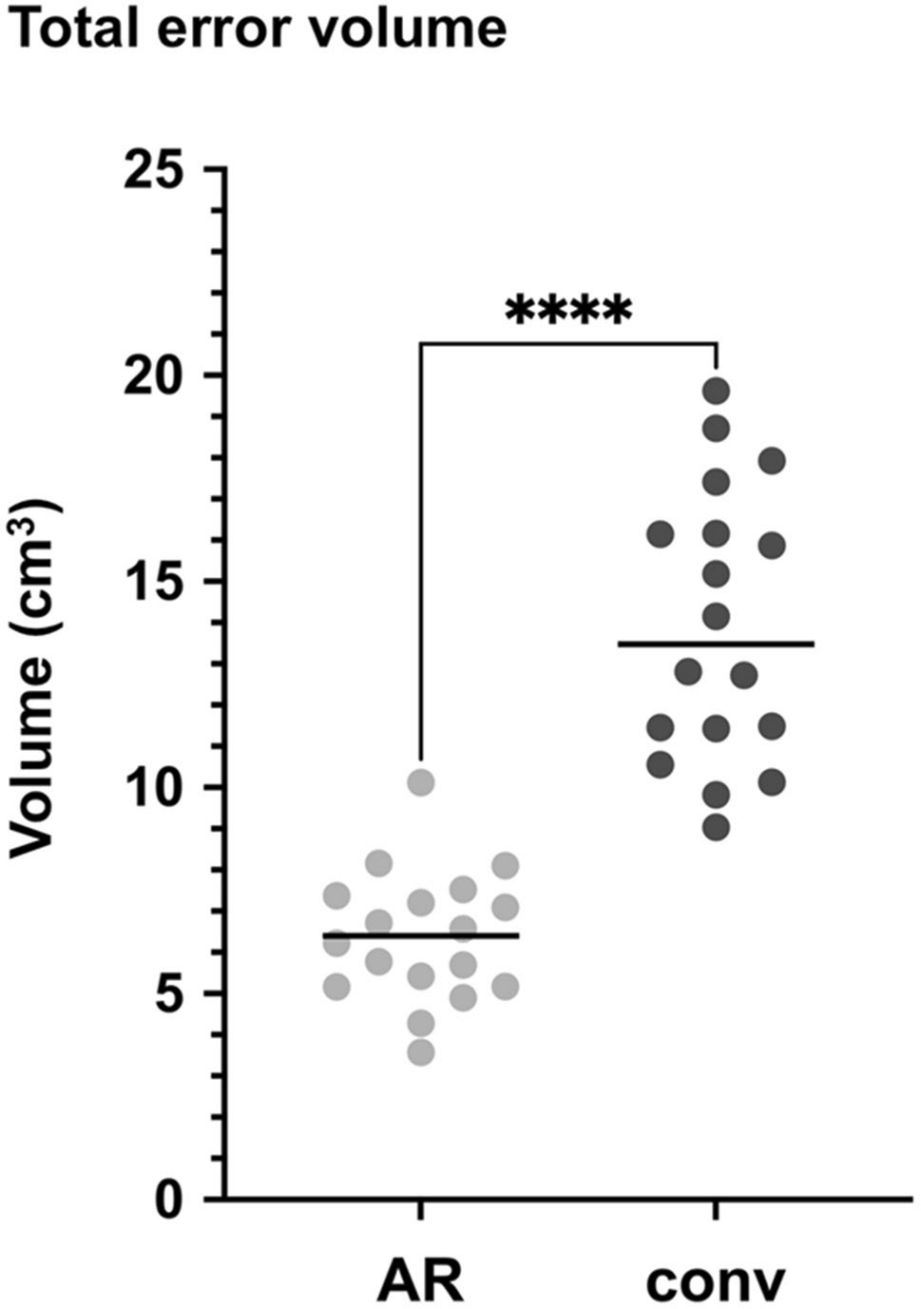
Total error volume of AR-assisted and conventionally shaped implants. Light gray dots, total error volumes (*E*_total_ = *E*_1_ + *E*_2_) of AR-assisted implants; dark gray dots, total error volumes (*E*_total_ = *E*_1_ + *E*_2_) of conventionally shaped implants; black horizontal lines, median values; ****, significant difference; AR, AR-assisted; conv, conventionally shaped

Compared with the surface profile of the original skull model, the median total error volume of the AR-assisted implants (*E*_total_ AR) was low and significantly reduced in comparison with that of the conventionally shaped implants (*E*_total_ conv), at 6.40 cm^3^ (IQR 2.24) versus (vs.) 13.48 cm^3^ (IQR 5.26), *p* < 0.0001 (Fig. 4). The median error volume overlapping the original skull model (*E*_1_) showed no significant difference between the AR-assisted and the conventionally shaped implants (3.78 cm^3^ (IQR 2.39) vs. 3.66 cm^3^ (IQR 6.63), *p* = 0.9626). The median error volume lacking in comparison with the original skull model (*E*_2_) showed a significant difference between the AR-assisted and the conventionally shaped implants (2.25 cm^3^ (IQR 2.73) vs. 8.84 cm^3^ (IQR 9.32), *p* = 0.0004). Detailed volumetric values are listed in Table 2.

**Table 2:** Volumetric analysis of AR-assisted and conventionally shaped implants

Implant number	*E*_1_ (volume in cm^3^)		*E*_2_ (volume in cm^3^)		*E*_total_ (volume in cm^3^)	
	AR	conv	AR	conv	AR	conv
1	3.30	3.92	3.90	5.11	7.20	9.03
2	5.09	0.42	1.13	19.20	6.22	19.62
3	6.10	2.63	2.00	15.30	8.10	17.93
4	2.69	2.22	4.68	10.50	7.37	12.72
5	4.66	4.11	5.46	6.44	10.12	10.55
6	3.20	2.16	2.49	14.00	5.69	16.16
7	5.75	17.20	1.77	1.51	7.52	18.71
8	1.84	1.37	1.73	14.50	3.57	15.87
9	6.29	3.70	0.42	6.43	6.71	10.13
10	1.39	1.33	2.89	10.10	4.28	11.43
11	4.71	10.50	0.46	0.98	5.17	11.48
12	4.45	6.14	1.32	8.01	5.77	14.15
13	3.67	3.15	1.23	9.66	4.90	12.81
14	2.32	8.65	4.25	2.80	6.57	11.45
15	3.89	9.37	1.27	0.45	5.16	9.82
16	2.43	2.58	4.66	12.60	7.09	15.18
17	4.51	3.61	3.65	13.80	8.16	17.41
18	2.38	9.78	3.04	6.36	5.42	16.14

Mean values (in cm^3^) of investigator 1 and investigator 2. *E*_*1*_, error volume overlapping the original skull model; *E*_*2*_, error volume lacking in comparison with the original skull model; *E*_*total*_, total error volume *E*_1_ + *E*_2_; *AR*, AR-assisted; *conv*, conventionally shaped

### Cosmetic appearance

**Fig. 5:**
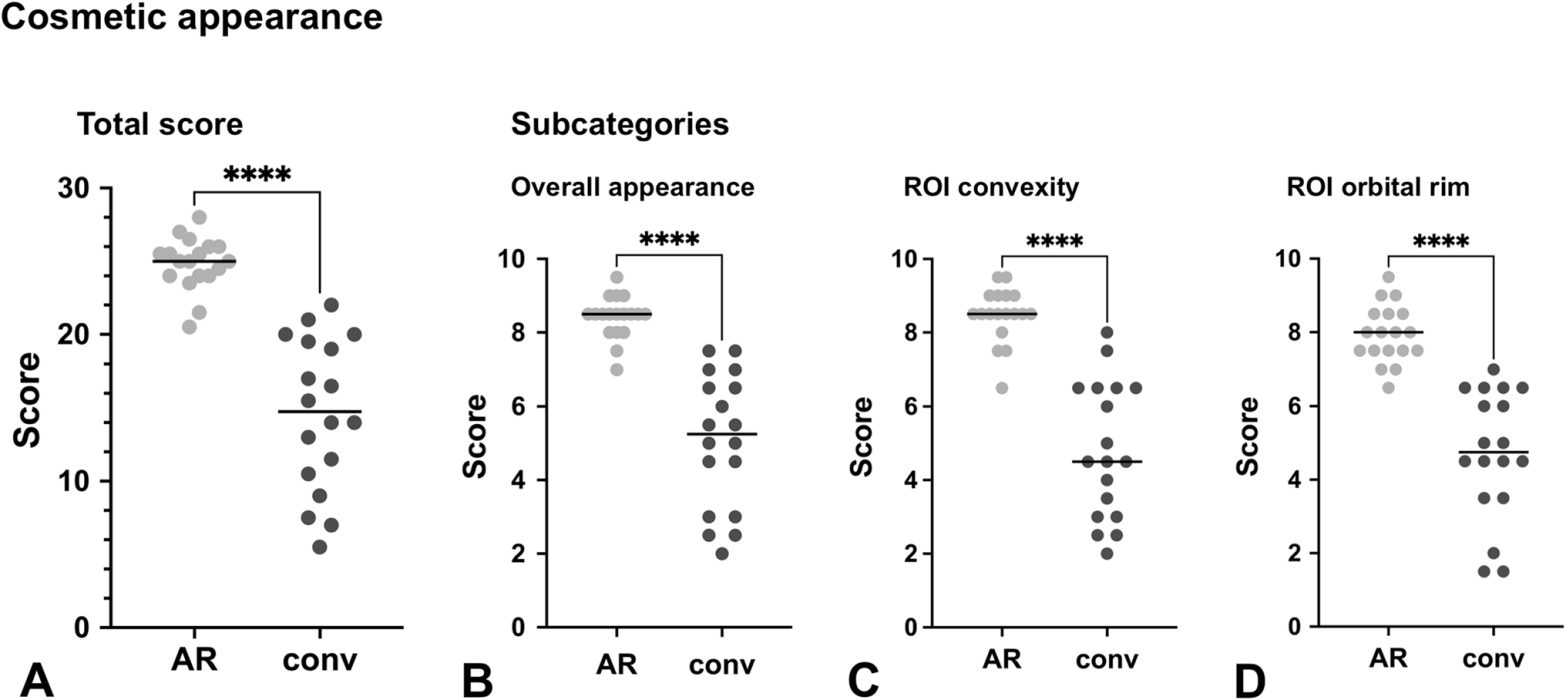
Cosmetic appearance of AR-assisted and conventionally shaped implants. Light gray dots, scores of AR-assisted implants; dark gray dots, scores of conventionally shaped implants; black horizontal lines, median values; ****, significant difference; AR, AR-assisted; conv, conventionally shaped. (**A**) Total scores of cosmetic appearance (sum of subcategories with minimum: 0 and maximum: 30); (B–D) Subcategory scores of cosmetic appearance (each with minimum: 0 and maximum: 10); (**B**) overall appearance; (**C**) ROI convexity, ROI convexity of the frontal tuberosity; (**D**) ROI orbital rim, ROI reconstruction of the orbital rim

The cosmetic appearance of the AR-assisted implants was rated as significantly better than that of the conventionally shaped implants. With a possible maximum total score of 30, the median total score of the AR-assisted implants was 25.00 (IQR 2.00) and that of the conventionally shaped implants was 14.75 (IQR 9.5), *p* < 0.0001 (Fig. 5). The AR-assisted implants also rated significantly better in all different subcategories of cosmetic appearance (overall appearance as well as the specific ROIs (convexity of the frontal tuberosity and reconstruction of the orbital rim)) (Fig. 5). The evaluation of the cosmetic results in detail is given in Table 3.

**Table 3:** Cosmetic appearance of AR-assisted and conventionally shaped implants

	Implant number	Overall appearance (score 0–10 points)	ROI convexity (score 0–10 points)	ROI orbital rim (score 0–10 points)	Total score (score 0–30 points)
AR	1	8.5	8.5	9	26
	2	8.5	9	7.5	25
	3	7	6.5	7	20.5
	4	8.5	8.5	7.5	24.5
	5	7.5	7.5	6.5	21.5
	6	9	9.5	8.5	27
	7	8	8	8	24
	8	8.5	8.5	8	25
	9	8	8.5	7.5	24
	10	9	9	7.5	25.5
	11	8.5	8.5	8	25
	12	8.5	7.5	7.5	23.5
	13	8.5	8.5	8.5	25.5
	14	9.5	9	9.5	28
	15	9	9.5	8	26.5
	16	8	9	7	24
	17	8.5	8.5	9	26
	18	8.5	8.5	8.5	25.5
conv	1	7.5	8	6.5	22
	2	3	2.5	3.5	9
	3	4.5	3.5	3.5	11.5
	4	5.5	5	5	15.5
	5	6.5	6.5	6	19
	6	2.5	3	2	7.5
	7	3	2.5	5	10.5
	8	5	4.5	4.5	14
	9	5	4.5	4.5	14
	10	7	6	7	20
	11	7.5	7.5	6	21
	12	5.5	6.5	4.5	16.5
	13	6	4.5	6.5	17
	14	7	6.5	6.5	20
	15	6.5	6.5	6.5	19.5
	16	4.5	4	4.5	13
	17	2.5	3	1.5	7
	18	2	2	1.5	5.5

Mean score values of investigator 1 and investigator 2. *ROI*, region of interest; *ROI convexity*, ROI convexity of the frontal tuberosity; *ROI orbital rim*, ROI reconstruction of the orbital rim; *AR*, AR-assisted; *conv*, conventionally shaped

## Discussion

Patient-specific CAD/CAM implants are increasingly used because of their better cosmetic results in comparison with, for instance, prefabricated titanium implants or freehand-shaped PMMA implants, particularly in patients with benign lesions who undertake normal life activities and do not require immediate surgery [1, 3, 14]. However, due to the rather high costs, these commercially manufactured patient-specific implants are not available to every patient in countries with limited healthcare resources. Therefore, efforts should be made to find more cost-efficient methods for achieving adequate cosmetic results. Especially in patients with benign lesions of the anterior skull base with involvement of the forehead or orbit, often middle-aged women, this appears to be important also in a sociocultural context [14, 27]. With regard to costs and infrastructure, the importance of a single-step surgical procedure also needs to be emphasized. Several authors describe a single-step surgical procedure for resection and reconstruction of benign craniofacial skull base lesions, however, through the use of cost-intensive implants previously fabricated based on virtual resection margins [10, 17, 28, 37]. Furthermore, this method becomes less suitable if resection margins need to be spontaneously extended intraoperatively, or for immediate reconstruction in malignant lesions or after trauma.

In the presence of a relatively intact bone flap that needs to be replaced, such as in resection of intraosseous meningiomas or calvarial metatases, an efficient and cost-effective method is described that has no special technical requirements. With this simple procedure, a PMMA implant is fabricated based on the bone flap to be replaced and its negative form, which appears to be an excellent method for selected cases, but seems less suitable for complex defects with craniofacial involvement or in cases where skull continuity is lost [21].

Recent publications report a highly interesting, cost-effective alternative for covering cranial defects based on 3D-printed molds, which were used to intraoperatively fabricate PMMA implants [15, 18, 22, 30, 34]. In cases without a suitable preoperative CT dataset for 3D printing, the authors describe how templates were prepared on the basis of the mirrored contralateral side [34]. This is also an interesting option in the context of the described AR-based method for cases in which osteolysis, hyperostoses, fractures, or tumors with disturbed surface continuity are present and therefore cannot serve as ground truth for the holographic projection. In our phantom study, these conditions were optimal because a regular skull surface was available as ground truth before insertion of the defect. Cosmetic results of these implants created on the basis of 3D-printed molds have been reported to be very encouraging; however, the appropriate technical conditions are required. In addition, this technique has usually been used thus far for skull defects without relevant involvement of the face or the orbital rim, typically after decompression craniectomy in patients with neurological impairments [15, 30]. However, this method also appears to be promising for the reconstruction of anatomically more complex defects and should be further developed, even because of its potentially low costs and wide availability.

In this context, AR holds high capability for future concepts. In spinal neurosurgery, AR-assisted procedures have been applied especially for screw placement in combination with navigation or robot assistance within the last decade [12, 23, 35]. Phantom studies and cadaver simulations have also demonstrated promising applications of AR in cranial neurosurgery with techniques for cranial biopsy or accurate catheter placement. This raises the potential for very precise minimally invasive procedures, which could perspectively be applied bedside with minor efforts [33, 36]. AR-assisted puncture of the gasserian ganglion for precise radiofrequency ablation in trigeminal neuralgia provides another interesting approach in minimally invasive interventional pain management [26]. For cranial neurosurgery, besides the applications of AR in phantom studies or for preoperative simulations and training, the first descriptions of intraoperative use can also be found (AR-assisted navigation) [9, 16, 18, 25]. The development of AR glasses provides another pioneering field, allowing the surgeon to move around in the room and view an object from all sides or even walk through it. To our knowledge, our study is the first to describe the use of AR glasses in fabricating PMMA implants with which to cover craniofacial defects.

Handling of the AR equipment was quite intuitive and after registration both hands were free to work on the implants. The AR image allowed adaptation of the implant to the holographic projection of the original skull model, which was accurately visualized even in complex regions. The evaluation of our participants reflects that the AR equipment being worn on the body was not experienced as disturbing and the entire AR handling was found to be easy to deal with, regardless of the individual expertise. The use of AR was definitely considered to be a benefit for implant fabrication. Participants mostly had the subjective impression of achieving good cosmetic results when using AR, and this usually correlated with satisfaction with results after finishing the implants. General satisfaction with cosmetic results of AR-assisted implants was high. During the fabrication of the conventionally shaped implants, subjective impressions varied about whether good results were achieved, and participants later were rather dissatisfied with the results. The manual registration of the real object and the holographic projection, which had to be readjusted for each run, was seen as problematic because it was time-consuming and a potential source of error. There is a need for optimization regarding the lack of an automatic registration mechanism. Nevertheless, additional manual adjustment should still be possible, since the automatic registration mechanisms may not yet guarantee the high accuracy that is required for skull base applications in particular [11].

Patient-specific CAD/CAM implants represent the gold standard for skull reconstruction. With these, only minimal deviations from the original surface profile and an excellent esthetic appearance can be assumed. Reports on less expensive implants manufactured with 3D-printed molds also describe very good cosmetic results, but an objective quantitative analysis, as performed in the current work by means of volumetry, cannot be found in any of these articles [15, 20, 22, 30].

With regard to cost calculation in a Western country, a price of at least 4000–5000 USD can be assumed for a patient-specific CAD/CAM implant made of PMMA, given the size and complexity of our study implant. Comparative data can be found in a recent Canadian publication on cost-effectiveness in cranioplasty [6]. Here, 1 min in the operating room (OR) was calculated to cost approximately 20 USD. In the current study, the time required for AR-assisted implant fabrication was 30–40 min per implant (manual registration process, preparation of the situs, PMMA fitting), resulting in 600–800 USD for additional OR time compared to prefabricated CAD/CAM implants. The estimated costs per implant for PMMA and other disposable materials in our study were 80–100 USD; and the one-time price for purchasing a Magic Leap is about 2300 USD. Thus, with established AR-assisted implant fabrication with regular use of the purchased Magic Leap, the costs per implant could probably be kept below 1000 USD, compared to several thousand USD of a prefabricated CAD/CAM implant.

Our presented method therefore offers the potential of a cost-effective and widely available single-step procedure for the treatment of complex, cosmetically challenging skull defects, even in the context of immediate reconstruction in malignant lesions or after trauma. Despite the limited number of implants, the statistical results seem to be promising. Compared with the ground truth, the AR-assisted implants deviated only slightly in volume (*E*_total_ = 6.40 cm^3^), whereas this was twice as much for the conventionally shaped implants (*E*_total_ = 13.48 cm^3^). The cosmetic appearance of the AR-assisted implants was confirmed to be very good by two blinded raters. Thus, with a maximum score of 30 to be achieved, the median score of the AR-assisted implants was high at 25.00.

There was no considerable prolongation of the procedure when AR was used. Manual registration took a few additional minutes, but the process of preparing the situs and shaping the implant was not significantly prolonged by the use of AR. This was also due to the fact that PMMA hardens relatively quickly and no subsequent optimization was performed. However, AR has the advantage that the implant can be flexibly adapted intraoperatively to previously undetermined resection margins and can be optimized afterwards as required.

### Limitations of the study

Although patient-specific CAD/CAM implants remain the gold standard, our results show that AR-assisted procedures hold the potential for excellent cosmetic results. However, our current work is a first approach in this field and has certain limitations. For example, the AR glasses have not yet been approved for intraoperative use but only as a viewing device during planning. A Brainlab navigation system is yet required for application, thus restricting the availability for countries with limited healthcare resources. Perhaps telemedicine could provide a solution that would allow the AR glasses to work online-driven and without the need for the entire navigation system to be on site. Naturally, it was not possible to blind the participants during the fabrication of the implants; thus, some bias may be implicated. By using experienced and less experienced surgeons and randomizing between the fabrication of the AR-assisted and the conventionally shaped implants, an attempt was made to minimize the bias within the results. Our limited number of nine participants and of each 18 AR-assisted and 18 conventionally shaped implants should also be mentioned. In order to maximize the validity, both the volumetric analysis and the evaluation of cosmetic outcomes were performed in a blinded fashion by two independent raters.

## Conclusions

Our experiments showed promising results regarding the possibilities of AR-assisted procedures for single-step reconstruction of craniofacial defects, both in terms of quantitative analysis by means of volumetry and in terms of multicomponent evaluation of cosmetic appearance. Although patient-specific CAD/CAM implants represent the gold standard in esthetic aspects, AR-assisted procedures hold a high capability in providing similar cosmetic results while offering the potential of an immediately and widely available, more cost-effective alternative.

## Supplementary Information

Below is the link to the electronic supplementary material.

Supplementary file1 (MOV 126380 KB)

## Data Availability

The datasets generated and analyzed during the current study are available from the corresponding author on reasonable request.

## Abbreviations

3D: three-dimensional
AR: augmented reality
CAD/CAM: computer-assisted design/manufacturing
CT: computed tomography
IQR: interquartile range
kV: kilovolt
mAs: milliampere-second
OR: operating room
PMMA: polymethylmethacrylate
ROI: region of interest
USD: United States dollar
vs.: versus
